# Case Report: Evaluating toxic encephalopathy from occupational 1,2-dichloroethane exposure: magnetic resonance imaging contributions

**DOI:** 10.3389/ftox.2025.1557995

**Published:** 2025-06-25

**Authors:** Jieru Wang, Tianzi Jian, Guangcai Yu, Baotian Kan, Wei Li, Xiangdong Jian

**Affiliations:** ^1^ Department of Critical Care Medicine, The 5th People’s Hospital of Jinan, Jinan, Shandong, China; ^2^ Department of Poisoning and Occupational Diseases, Emergency Medicine, Qilu Hospital of Shandong University, Cheeloo College of Medicine, Shandong University, Jinan, Shandong, China; ^3^ Department of Nephrology, The Affiliated Hospital of Shandong University of Traditional Chinese Medicine, Jinan, Shandong, China; ^4^ Department of Geriatric Medicine, Department of Nursing, Qilu Hospital of Shandong University, Cheeloo College of Medicine, Shandong University, Jinan, Shandong, China

**Keywords:** 1,2-dichloroethane, toxic encephalopathy, occupational exposure, cerebral edema, magnetic resonance imaging, clinical features

## Abstract

**Background:**

1,2-Dichloroethane is a commonly used industrial solvent. Acute or subacute occupational exposure can cause toxic encephalopathy; however, long-term changes in brain imaging are not frequently documented.

**Case presentation:**

A 39-year-old woman developed dizziness and forgetfulness 9 days after performing glue coating. Her symptoms improved significantly after a 7-day break from work. However, after resuming work for 3 days, she returned with dizziness, headache, and anxiety. Brain magnetic resonance imaging (MRI) showed extensive edema and diffuse abnormal signal intensities in the cerebellar dentate nucleus, basal ganglia, and bilateral cerebral white matter. She was treated with salvianolate injection, magnesium isoglycyrrhizinate, and neurotrophic therapy. Two weeks after admission, her symptoms improved significantly, except for mild uncoordinated walking. The range of abnormal MRI signals remained consistent with previous findings. She was discharged the following day. She experienced worsened headache 3 days later. Computed tomography revealed diffuse cerebral edema. Despite treatment with mannitol, her headache rapidly worsened and was accompanied by nausea, vomiting, hypertension, bradycardia, and dyspnea, ultimately leading to unconsciousness. Follow-up MRI showed findings similar to the previous scan, except that the apparent diffusion coefficient (ADC) sequence had changed from hypointense to hyperintense. Shortly after the MRI examination, she experienced respiratory arrest. Unfortunately, she died 32 days after her initial admission due to severe cerebral injury and infection.

**Conclusion:**

Occupational exposure to 1,2-dichloroethane can lead to toxic encephalopathy, presenting as diffuse progressive cerebral edema. This case shows that brain imaging findings may not always correlate with the patient’s clinical condition, so careful monitoring is essential.

## 1 Introduction

1,2-Dichloroethane (DCE) is a toxic industrial solvent that can cross the blood-brain barrier (BBB) and cause significant neurological damage (Chen et al., 2015; Dang et al., 2019). Although the acute effects of DCE exposure are well-documented, long-term changes observed via brain imaging are rarely reported. This study aimed to elucidate the dynamic clinical features and brain magnetic resonance imaging (MRI) findings of DCE-induced toxic encephalopathy, with a particular focus on the often-overlooked long-term imaging changes in the literature.

## 2 Case presentation

**FIGURE 1:**
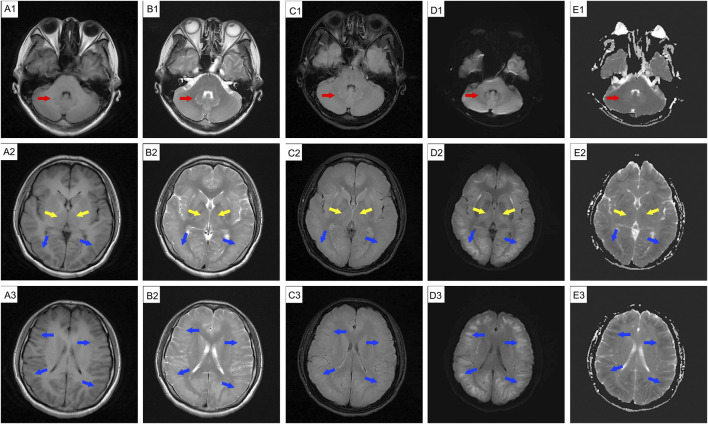
MRI findings of a 39-year-old female glue-coating worker with headache and dizziness after admission (29 August 2024). MRI revealed extensive edema and abnormal signal intensity is observed in the cerebellar dentate nucleus (red arrow), basal ganglia (yellow arrow), and bilateral cerebral hemisphere white matter (blue arrow). Hyperintense regions are present on T2WI **(B1-B3)**, T2-FLAIR **(C1-C3)**, DWI **(D1-D3)**, and ADC (**E1**, dentate nuclei); hypointense areas are noticeable on T1WI **(A1-A3)** and ADC **(E2-E3)**. MRI: magnetic resonance imaging.

**FIGURE 2:**
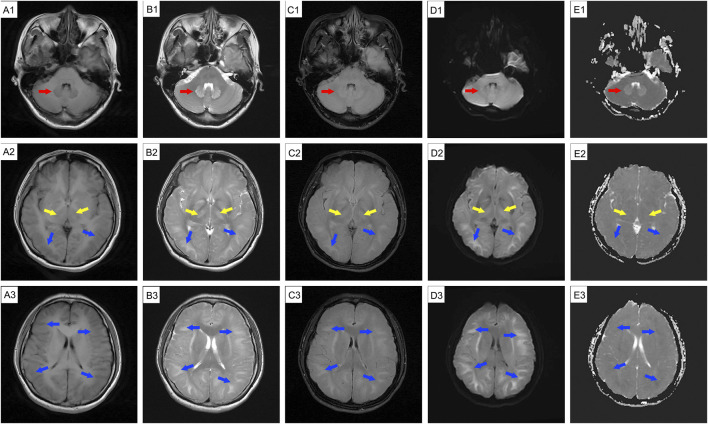
Follow-up cranial MRI before discharge (10 September 2024) showing abnormal signal intensity. The patient experienced mild fatigue and intermittent headache. MRI revealed abnormal signal intensity is observed in the cerebellar dentate nucleus (red arrow), basal ganglia (yellow arrow), and bilateral cerebral hemisphere white matter (blue arrow); MRI also reveals swollen gyri, shallow sulci, and shrunken ventricles. Hyperintensities are present on T2WI **(B1-B3)**, T2-FLAIR **(C1-C3)**, DWI **(D1-D3)**, and ADC (**E1**, dentate nuclei) images, while hypointensities can be seen on T1WI **(A1-A3)** and ADC maps **(E2-E3)**. MRI: magnetic resonance imaging.

**FIGURE 3:**
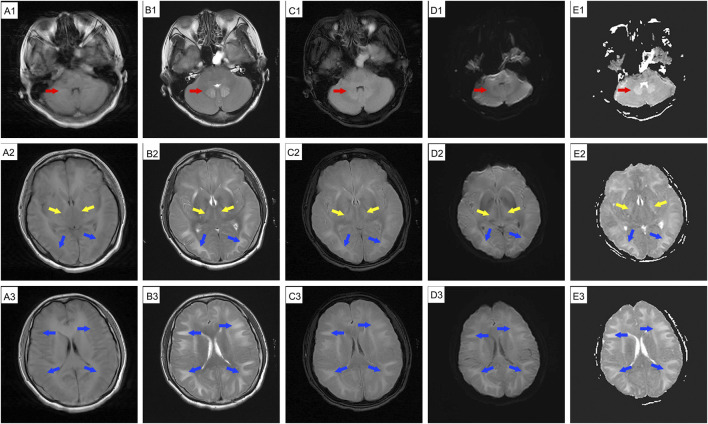
Emergency cranial MRI before respiratory arrest (19 September 2024), during cerebral herniation, reveals swollen gyri, shallow sulci, and shrunken ventricles across all imaging sequences. Abnormal signal intensity in the cerebellar dentate nucleus (red arrow), basal ganglia (yellow arrow), and bilateral cerebral hemisphere white matter (blue arrow). Hyperintensities are present on T2WI **(B1-B3)**, T2-FLAIR **(C1-C3)**, DWI **(D1-D3)**, and ADC **(E1-E3)** images, and hypointensities are present on T1WI **(A1-A3)**. MRI: magnetic resonance imaging.

**FIGURE 4:**
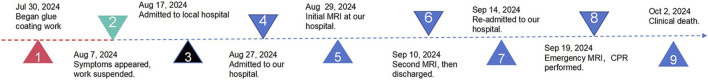
The patient’s treatment timeline.

A 39-year-old female glue-coating worker developed mild dizziness and forgetfulness after 9 days of continuous work. Initially, she visited a local community hospital, where she was treated for cervical spondylosis. After a 7-day break from work and treatment, her dizziness improved. Three days after returning to work, her condition deteriorated, presenting new symptoms of headache, gastrointestinal distress, and increased anxiety. She presented to a local central hospital, where MRI was used to diagnose toxic or metabolic encephalopathy. The following day, she was transferred to our hospital. She had no history of hypertension, diabetes, long-term hunger, chronic medication use, long-term alcohol consumption, trauma, mental disorders, or episodes of headache or dizziness. She also had no history of long-term toxic exposure, having only recently worked in transparent tape coating, where she operated a coating machine for 12 h a day. Despite wearing disposable masks and regular overalls, she frequently reported smelling a chloroform-like odor. Upon admission, she reported headache, dizziness, forgetfulness, and anxiety. Physical examination was unremarkable except for mild uncoordinated walking. Basic laboratory tests and blood toxicology screenings for DCE and other solvents were normal. Investigations revealed that the glue to which the patient was exposed contained DCE, methanol, and xylene. She received treatments including salvianolate injection, magnesium isoglycyrrhizinate, mecobalamin, vitamin B1, and aspirin. By the next day, her headache had significantly resolved, but she continued to experience mild dizziness and fatigue. Brain MRI revealed extensive edema and diffuse abnormal signal intensities in the cerebellar dentate nucleus, basal ganglia, and bilateral cerebral hemisphere white matter, confirming toxic encephalopathy (Figure 1). Fourteen days after admission, she reported slight fatigue and mild uncoordinated walking. Brain MRI results are shown in Figure 2, where the primary change is that the apparent diffusion coefficient (ADC) sequence of the cerebellar dentate nucleus changed from hypointense to hyperintense signals. She was discharged the following day. Three days after discharge, her headache worsened, prompting her to return to our department the following day. Her vital signs were stable. Her laboratory test results were unremarkable. Brain computed tomography revealed diffuse swelling in bilateral cerebral hemispheres. Treatment was resumed as previously described, except for mannitol. On the afternoon of the second day post-return, her headache worsened with nausea and vomiting and was temporarily alleviated by intravenous mannitol. On the third morning, she developed hypertension, bradycardia, dyspnea, and apathy, necessitating mannitol administration. Compared with previous scans, brain MRI revealed that the primary change was hypointense to hyperintense signals in the ADC sequences (Figure 3). Unfortunately, she experienced respiratory and cardiac arrest immediately after MRI and received cardiopulmonary resuscitation. Subsequently, she was intubated and mechanically ventilated due to weak breathing. Her family refused lateral ventriculopuncture and other surgical treatments. The day after resuscitation, laboratory tests revealed elevated levels of liver enzymes (ALT: 259 U/L, AST: 254 U/L), creatine kinase (CK: 459 U/L), and cardiac troponin (cTnT: 10780.43 ng/mL). Sputum and blood cultures returned negative. Electrocardiography revealed sinus tachycardia (rate: 135 beats/min), and bedside ultrasonography revealed heart failure. On the 32nd day after admission, her cardiac function improved, but she remained in a persistent deep coma and developed a severe infection. Due to financial concerns, her family declined further treatment, and she was discharged. She subsequently died. Her treatment timeline is shown in Figure 4.

## 3 Discussion

DCE, also known as 1,2-ethylenedichoride, dichloroethylene, or ethylene dichloride, is a colorless oily liquid with a chloroform-like odor and sweet taste (Dang et al., 2019; Xiang et al., 2023). It is commonly used as an industrial solvent and in the formulation of adhesives (Liu et al., 2019). It is highly toxic, and can be absorbed through the respiratory tract, gastrointestinal tract, and skin, leading to central nervous system, liver, kidney, and heart damage (Chen et al., 2015; Dang et al., 2019; Huang et al., 2020). The clinical features often include headache, dizziness, forgetfulness, nausea, vomiting, fatigue, apathy, excessive sleepiness, speech disturbances, ataxia, delirium, and myoclonus (Dang et al., 2019; Liu et al., 2019). Occupational exposure occurs mainly through vapor inhalation, with frequent reports of subacute toxic encephalopathy (Liu et al., 2019). DCE can easily cross the BBB and accumulate in lipophilic tissues, especially brain tissue (Zeng et al., 2019), leading to cytotoxic edema and white matter demyelination (Zhou et al., 2015). Intracranial hypertension, a typical clinical feature of DCE-induced toxic encephalopathy, is generally linked to cerebral edema (Xiang et al., 2023; Zhan et al., 2011). Cerebral edema is classified into vascular and cytotoxic forms. Cytotoxic edema primarily involves excess fluid accumulation in the intracellular compartments, primarily during the acute stage of DCE exposure (Chen et al., 2015; Zhong et al., 2023). These hypotheses may involve BBB damage, energy metabolism (Huang et al., 2020; Yu et al., 2024), AQP4 inhibition (Zhong et al., 2022), Ca2+ overload, reactive oxygen species, free radicals, and neurotransmitter changes (Xiang et al., 2023). Vascular brain edema evolves from cytotoxic edema that occurs in the subacute stage and arises from the compression of blood vessels by edematous cells (Xiang et al., 2023). White matter demyelination is usually a secondary injury that manifests as astrocyte necrosis (Zhou et al., 2015). Brain MRI is vital for identifying the types of brain edema. Vasogenic edema appears to have a high intensity on diffusion-weighted imaging (DWI) and ADC, whereas cytotoxic edema appears to have a high and low intensity on DWI and ADC, respectively (Chen et al., 2015; Zhan et al., 2011). In our case, diffuse cerebral edema was evident in all imaging sequences, manifesting as swollen gyri, shallow sulci, and diminished ventricles; there were abnormal lesions in the white matter in both cerebral hemispheres, the basal ganglia, and the dentate nucleus of the cerebellum, corresponding with previous reports (Chen et al., 2015). The patient’s persistent mild ataxia and unsteady walking may have been related to cerebellar vermis damage (Huang et al., 2020). The initial brain MRI revealed cellular edema (Figure 1, high intensity on DWI and high or low intensity on ADC) with mild clinical features of intracranial hypertension. Two weeks later, a repeat MRI showed vasogenic edema (Figure 2, high-intensity DWI and ADC). Despite this patient not using osmotic-dehydrating agents, her symptoms improved significantly. Unfortunately, she subsequently developed severe intracranial hypertension, leading to respiratory and cardiac arrest.

Early diagnosis and management of cerebral edema are crucial for treating DCE-induced toxic encephalopathy, as the severity of cerebral edema, particularly intracranial hypertension, is closely associated with the worsening of the patient’s condition (S. Chen et al., 2015; Dang et al., 2019; Liu et al., 2019). In this case, the patient’s death resulted from the oversight of cerebral edema during treatment. The patient developed cerebral herniation despite late-stage mannitol treatment, likely due to inadequate early management of the progressive cerebral edema. Lumbar puncture evaluates the severity of intracranial hypertension, alleviates pressure by draining excess cerebrospinal fluid, and helps to identify infections or inflammation that contribute to increased pressure. Surgical intervention after cerebral herniation is an effective measure to reduce intracranial hypertension and is likely to improve the prognosis (Zhou et al., 2015). The early and long-term use of glucocorticoids and dehydrating agents is usually effective (Chen et al., 2015; Dang et al., 2019). However, the brain edema was progressive, and MRI findings were not always consistent with the clinical features. Timely and repeated brain MRI, along with diffusion-weighted imaging and intracranial pressure monitoring, are essential for effectively monitoring symptom onset. In addition, familiarity with the toxicological characteristics of DCE, along with occupational exposure factors and protective measures (Table 1), is essential for the early detection of occupational DCE poisoning and for reducing its incidence.

**TABLE 1:** Physical properties, toxicokinetic characteristics, occupational exposure factors, and protective measures of DCE.

Items	Descriptions
Physical Properties	Molecular weight: 98.96 g/mol; melting point: −34.0°C; boiling point: 83.5°C; density: 1.25 g/cm^3^ (20 °C). Colorless liquid with a sweet, pungent odor, slightly soluble in water and miscible with organic solvents (e.g., alcohols, ethers, benzene). Moderate volatility and can form concentrated vapors in air.
Toxicokinetics (Absorption, Distribution, Metabolism, Excretion) (Agency for Toxic Substances and Disease Registry ATSDR Toxicological Profiles, 2024; Chen et al., 2019; Sweeney et al., 2008)	DCE is effectively absorbed through inhalation, oral intake, or skin contact. It reaches equilibrium blood levels in animals within 2–3 h of inhalation, 15–60 min of oral intake, and 1–2 h of dermal exposure. Once absorbed, DCE is widely distributed, especially in adipose tissue (7–17 times higher than in blood), in a dose-dependent manner, without bioaccumulation. It is metabolized via CYP oxidation and glutathione conjugation, producing metabolites like 2-chloroacetaldehyde, 2-chloroethanol, and 2-chloroacetic acid. Glutathione conjugation is more significant at higher doses (5–10 µg/mL blood levels). Final metabolites, thiodiglycolic acid and thiodiglycolic acid sulfoxide, are excreted in urine (84%). DCE and its metabolites are rapidly eliminated, with nearly complete excretion within 48 h of acute exposure in animal studies.
Toxicology Diagnostics (Agency for Toxic Substances and Disease Registry ATSDR Toxicological Profiles, 2024)	Blood levels of DCE and its metabolites (2-chloroacetaldehyde, 2-chloroethanol, and 2-chloroacetic acid) and urinary metabolites (thiodiglycolic acid and thiodiglycolic acid sulfoxide) are key diagnostic indicators within 48 h of exposure. After 48 h, due to rapid elimination (96%), the above tests become unreliable. Diagnosis post-48 h depends on exposure history, neurological findings, and liver/kidney lab results.
Occupational Exposure Factors (Malta et al., 2024)	The improper work environment, inadequate use of safety equipment, unsafe working conditions, individual issues such as fatigue, stress, insufficient training, and non-compliance with safety protocols.
Protective and Preventive Measures (Agency for Toxic Substances and Disease Registry ATSDR Toxicological Profiles, 2024; Chen et al., 2015)	Mark warning signs for toxic substances. Ensure ventilation with fume hoods or exhaust systems. Provide appropriate personal protective equipment, including gloves, goggles, and respirators. Conduct training on handling, storage, and disposal, and educate workers on exposure symptoms. Adopting safer alternatives to mitigate risks. Offer rest breaks to prevent fatigue and stress, and implement health surveillance with routine exams.

## Data Availability

The original contributions presented in the study are included in the article/supplementary material, further inquiries can be directed to the corresponding authors.
